# The wastewater micropollutant carbamazepine in insectivorous birds—an exposure estimate

**DOI:** 10.1007/s00216-022-04117-0

**Published:** 2022-05-17

**Authors:** Anna-Jorina Wicht, Katharina Heye, Anja Schmidt, Jörg Oehlmann, Carolin Huhn

**Affiliations:** 1 Institute of Physical and Theoretical Chemistry, Eberhard Karls Universität Tübingen, Tübingen, Germany; 2 Department Aquatic Ecotoxicology, Goethe-Universität Frankfurt, Frankfurt, Germany; 3 Bayerisches Landesamt für Umwelt, Augsburg, Germany

**Keywords:** QuEChERS, Metamorphosis, Body burden, *Chironomus riparius*, Habitat transfer

## Abstract

Insects with aquatic life stages can transfer sediment and water pollutants to terrestrial ecosystems, which has been described for metals, polyaromatic hydrocarbons, and polychlorinated chemicals. However, knowledge of the transfer of aquatic micropollutants released by wastewater treatment plants is scarce despite some preliminary studies on their occurrence in riparian spiders. In our study, we address a major analytical gap focusing on the transfer of the micropollutant carbamazepine from the larvae to the adult midges of *Chironomus riparius* using an optimized QuEChERS extraction method and HPLC–MS/MS applicable to both life stages down to the level of about three individuals. We show that the uptake of carbamazepine by larvae is concentration-dependent and reduces the emergence rate. Importantly, the body burden remained constant in adult midges. Using this information, we estimated the daily exposure of insectivorous tree swallows as terrestrial predators to carbamazepine using the energy demand of the predator and the energy content of the prey. Assuming environmentally relevant water concentrations of about 1 μg/L, the daily dose per kilogram of body weight for tree swallows was estimated to be 0.5 μg/kg/day. At places of high water contamination of 10 μg/L, the exposure may reach 5 μg/kg/day for this micropollutant of medium polarity. Considering body burden changes upon metamorphosis, this study fills the missing link between aquatic contamination and exposure in terrestrial habitats showing that wastewater pollutants can impact birds’ life. Clearly, further analytical methods for biota analysis in both habitats are urgently required to improve risk assessment.

**Graphical abstract:**

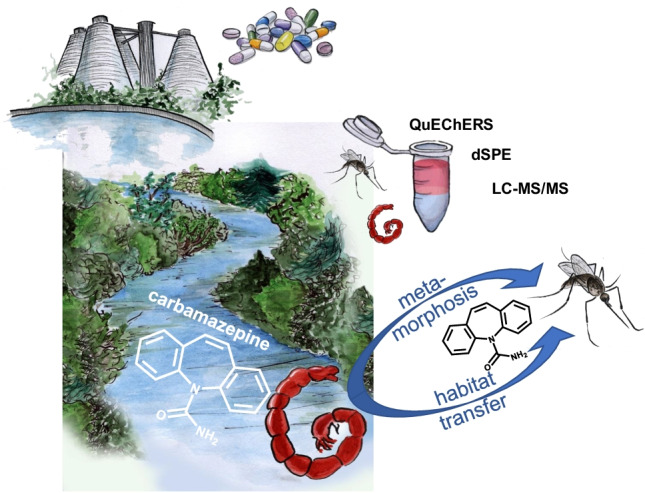

## Introduction

Pharmaceuticals released into surface waters from insufficient removal during wastewater treatment may be taken up and bioaccumulated by aquatic invertebrates [1]. Upon metamorphosis, a transfer to terrestrial life stages is possible. The awareness for the biologically mediated transfer of contaminants from streams and lakes to the terrestrial ecosystem increases [2] and was often assessed via macroinvertebrates or riparian spiders, e.g., for polychlorinated biphenyls (PCBs) [3–5] and metals ([1], Kraus and Pomeranz in [2]). Studies showed that bioaccumulation of metals in insects is metal- and concentration-specific, with most metals being eliminated via feces, including the first adult excrement (meconium) with elimination rates up to 90% [6, 7]. In contrast, exposure to polyaromatic hydrocarbons (PAHs) resulted in 2.9-fold higher concentrations in larvae than in adults [7]. PCBs [7, 8] and polychlorinated pesticides [9] were also studied with residue findings for wildlife insects in their aquatic life stages, in the food of birds, in birds (mainly tree swallows, *Tachycineta bicolor*), and in riparian spiders.

Insect-mediated contaminant flux of micropollutants was addressed only recently for 98 pharmaceuticals quantified in aquatic invertebrates, most of them with a terrestrial adult life stage, and riparian spiders [1]. The authors identified a data gap with regard to transfer rates upon metamorphosis. This gap can only be closed when intensifying biota analysis for different habitats. We here try to fill this gap for the anti-epileptic drug carbamazepine which is detected in surface waters and effluents in concentrations of 84–790 ng/L in Germany [10] and worldwide up to 8 μg/L in surface waters [11] due to its high prescription rates. Chironomids were used as model organism forming the major prey for many birds [12, 13]. They are ideal sentinel organisms to assess sediment and aquatic contamination due to their high species richness [14]. In the UK, carbamazepine was detected at low nanograms per gram of concentrations in gammarids sampled in streams [15]. It is frequently detected in aquatic larval insects with emergent adult life stages and riparian spiders (more than 75% of all samples), however, at low nanograms per gram concentrations [1]. Species abundance changed with increasing carbamazepine concentrations in Indian rivers [16]. Ecotoxicological studies revealed effects on invertebrate’s growth, mortality, and emergence [17–19].

We here investigate the changes in carbamazepine body burden upon metamorphosis in larvae and adult midges of *Chironomus riparius*. Quantification was achieved with a new miniaturized QuEChERS (Quick Easy Cheap Effective Rugged and Safe) extraction for larvae and adult midges. We estimate the exposure of insectivorous tree swallows via dietary data, energy requirements of the predator, and energy resources of the prey based on the trophic transfer from aquatic to terrestrial ecosystems.

## Materials and methods

### Reagents, chemicals, and consumables

HPLC solvents methanol hypergrade LC–MS (Chromasolv), water hypergrade LC–MS (Chromasolv), acetonitrile LC–MS grade (Chromasolv) and formic acid (98%, eluent additive for LC–MS), carbamazepine for analysis, carbamazepine^*13*^*C*_*6*_ (^*13*^*C present in one benzene ring*), sodium chloride, and magnesium sulfate were purchased from Sigma-Aldrich (Steinheim, Germany). PSA bulk sorbent and C18 bulk sorbent were from Agilent Technologies (Waldbronn, Germany). Carbamazepine (chemical purity > 99%) for exposure tests, dimethyl sulfoxide (DMSO, purity: 99.5%), and the micro-homogenizer PP were delivered by Carl Roth (Karlsruhe, Deutschland). PTFE syringe filters 0.45 μm, 3 mm, were supplied by Macherey–Nagel (Düren, Germany).

### Biological exposure tests

A chronic life cycle toxicity test was performed according to OECD test guideline 219 (*Sediment–water chironomid toxicity using spiked water*). For each of the exposure treatments, 600-mL glass beakers were filled with 400 mL of M4 medium [20] and 120 g sediment, consisting of 99% (w/w) quartz sand (washed and heated to 200 °C), 0.5% (w/w) stinging nettle (*Urtica dioica*; particle size < 0.5 mm; Caelo, Hilden, Germany), and black alder leaves (*Alnus glutinosa* fall foliage; particle size < 0.5 mm). Carbamazepine was added to each of the test vessels at nominal exposure concentrations of 0.025, 0.05, 0.1, 0.2, 0.4, 0.8, 1.6, and 3.2 mg/L. As DMSO was used as solvent at a final concentration of 30 μL/L, a negative and solvent control had to be included in the experiment. The test vessels were gently aerated with Pasteur pipettes for 5 days before 20 chironomid larvae (< 24 h old) were added to each vessel. Larvae were fed three times per week with 0.25 mg/larvae/day of TetraMin in the first and last weeks. In between, the food portion was increased to 0.5 mg/larva/day. The experiment and culture were kept at 20 °C ± 1 °C and a light to dark cycle of 16:8 h.

Each of the treatments was set up in seven replicates. One replicate was used for analytical measurements of the exposure conditions. Two replicates were used for measurements of the internal concentrations. For analysis, larvae were removed from the vessels 10 days after insertion and transferred to control medium void of carbamazepine. Twenty-four hours later, larvae were frozen in liquid nitrogen and stored at – 80 °C until chemical analysis. The last four replicates were used to determine the emergence rate and internal concentrations of adult midges. Emerged midges were removed from the test vessels before they were killed by freezing to – 80 °C.

#### HPLC–MS/MS method

Samples were analyzed with HPLC–MS/MS as described in literature for carbamazepine residues in gammarids, fish, earthworm, and sediment extracts [21–24]. To account for matrix effects and analyte recovery during workup, an isotopically labeled internal standard was used for quantification. MS/MS (positive ionization mode) was used in order to enhance selectivity and signal to noise ratio.

A 1260 Infinity LC system coupled to a 6550 iFunnel QTOF HPLC–MS/MS system (Agilent Technologies, Waldbronn, Germany) was used. Sample aliquots of 10 μL were injected onto a Zorbax Eclipse Plus C18 column (2.1 × 150 mm, 3.5 µm, narrow bore, Agilent Technologies, Waldbronn, Germany) at a column temperature of 40 °C. A jet stream electrospray ionization (ESI) source was operated with a nebulizer pressure of 35 psig; drying gas temperature of 160 °C, at a flow rate of 16 L/min; and a fragmentor voltage of 360 V. In the positive ionization mode, capillary voltage was set to − 4000 V, skimmer voltage to 65 V, and nozzle voltage to − 500 V. The mass range was 100–1000 m/*z* with a data acquisition rate of 1 spectrum/s. For MS/MS spectra, the acquisition time was set to 200 ms/spectrum and the masses (*m*/*z* = 243.1236 and 237.1022) were isolated in a range of *m*/*z* = 4 in a retention time window of 11 ± 1 min. The MS/MS mode of the QTOF was optimized with respect to fragmentation voltage (300–400 V), collision energy (20–40 V), nozzle voltage (400–500 V), and octopole voltage (700–800 V). Best results were achieved for a collision energy of 24 V and a fragmentation voltage of 360 V in the positive ESI mode. With these parameters, the most abundant and dominant fragment ion was formed by loss of the amide group to the ion of *m*/*z* 194.097.

The sheath gas temperature was 325 °C with a flow rate of 11 L/min. For internal calibration, purine and HP0921 (Agilent Technologies, Waldbronn, Germany, *m*/*z* = 121.0508, 922.0097) were used. A gradient elution at a flow rate of 0.3 mL/min using water, containing 0.1% formic acid, and methanol was applied. The initial content of 95% water was decreased after 1 min to 5% water over 7 min and after another 7 min at 5% increased to 95% water over 0.5 min. Data analysis was performed with MassHunter software (Agilent Technologies, Waldbronn, Germany).

#### Analysis of exposure medium samples

Carbamazepine concentration in filtered samples of the exposure medium was analyzed at the beginning of the experiment at days 0, 14, and 28 (at the end of the exposure experiment). Samples were stored at − 20 °C. Aliquots of 2 mL were centrifuged for 3 min at 10,000 rpm, filtered and analyzed by HPLC–MS/MS. The water concentrations at days 0 and 14 were 63 ± 5% of the nominal concentrations, presumably due to sorption to sediment. At day 28, the concentrations further decreased to 50 ± 6% of the nominal concentration, indicating minor degradation processes. This process was pronounced for higher exposure concentrations, especially for 1.6 and 3.2 mg/L of carbamazepine. However, during the exposure, the concentrations were stable between 50 and 63% of the nominal concentration.

#### Sample preparation and analysis of biota samples

For the quantification of internal concentrations during the exposure study, 3–40 adult midges or 1–15 larvae were pooled (depending on the mortality rate) and two analytical replicates were processed in parallel. No carbamazepine was detected in larvae from the negative control.

For calibration in the matrix and method validation, samples were spiked with carbamazepine after homogenization and before extraction. To investigate signal suppression, carbamazepine and the internal standard were added to the final extract before injection to HPLC–MS at a final concentration of 20 μg/L.

In the final QuEChERS protocol, 25 mg of frozen larvae was homogenized in liquid nitrogen with a micro-homogenizer. Isotopically labeled internal standard carbamazepine^*13*^*C*_*6*_ in methanol was added resulting in a final concentration of 20 μg/L in 0.5 mL extract. After 1 h at room temperature, 0.5 mL acetonitrile and 0.5 mL water were added. For extraction, samples were shaken with a vortex device for 30 s, and 25 mg sodium chloride and 75 mg anhydrous MgSO_4_ were added and the sample was shaken for 30 s. After 3 min of centrifugation at 10,000 rpm, 0.4 mL of the organic acetonitrile phase was recovered.

The necessity of a cleanup of biota extracts by dispersed solid-phase extraction (dSPE) was investigated comparing results for raw extracts and cleanup using different SPE materials. (a) For the analysis of raw extracts, the organic layer was evaporated to dryness and the residue resolved in 0.25 mL methanol and filtered for analysis. (b) For the cleanup by dSPE, the organic layer was transferred to an Eppendorf tube containing dSPE sorbent. Three sorbents were tested for dSPE: (1) 12 mg PSA and 90 mg anhydrous MgSO_4_; (2) 12 mg C18 (non-endcapped) and 90 mg anhydrous MgSO_4_; (3) 12 mg PSA, 12 mg C18, and 90 mg anhydrous MgSO_4_. The sample was shaken for 30 s. After centrifugation for 3 min at 10,000 rpm, the acetonitrile phase was evaporated to dryness in a stream of nitrogen and the residue was reconstituted in 0.25 mL methanol. After filtration, samples were filtered using PTFE filters and analyzed by HPLC–MS/MS.

For calibration when analyzing biota samples, three replicates for each concentration level were extracted and purified with dSPE independently. Samples were spiked with 1, 2, 5, 10, 20, and 40 μg/L adding the internal standard at a concentration of 20 μg/L.

#### Data analysis

MassHunter Workstation software quantitative and qualitative analyses both version B.06.00 (Agilent Technologies, Waldbronn, Germany) were used for analysis. The retention time of carbamazepine was 11.2 min in scan mode with target *m*/*z* = 237.1022 ± 100 ppm (carbamazepine) and *m*/*z* = 243.1236 ± 100 ppm (isotopically labeled carbamazepine^*13*^*C*_*6*_). The transitions 237.1022 → 194.0971 (carbamazepine) and 243.1226 → 200.1171 (isotopically labeled carbamazepine^*13*^*C*_*6*_) were used in the MS/MS mode.

Measured values were tested for normal distribution by Shapiro–Wilk-test with Origin 9.1 (OriginLab, Northampton, USA) at a level of 0.05. If normal distribution was proven, significant differences of variances were tested with a one-way ANOVA using the software Origin 9.1 at a level of 0.05. The same software was also used for linear regression and fitting.

Signal suppression was calculated by the signal area of extracts spiked after extraction and before injection compared to the signal area of the standard in methanol at the same concentration.

$$\mathrm{Signal \, suppression \, (\%)= \frac{area (CBZ \, in \, extract)}{area(CBZ \, in \, MeOH)} \times 100}$$

Recovery was calculated by comparing the signal area in extracts spiked before the extraction to the one in extracts spiked prior to the measurement.

$$\mathrm{Recovery \, (\%)= \frac{area (spiked \, before \,extraction)}{area(spiked \, before \, measurement)} \times 100}$$

As the signal intensity of carbamazepine was always 1.18 times the signal intensity of the internal standard at the same concentration, possibly due to different ionization efficiencies, signal areas were corrected prior to the concentration calculation.

The bioconcentration factor BCF was calculated as the ratio of the carbamazepine concentration in biota versus its concentration in the medium.

$$\mathrm{BCF\left(\frac{L}{kg}\right)= \frac{concentration \, in \, biota ( \frac{mg}{kg} )}{concentration \, in \, medium ( \frac{mg}{L} )}}$$

## Results

### Development of the extraction protocol for larvae and midges

Sample extraction was based on a QuEChERS [25] method adapted from procedures optimized for carbamazepine extraction from earthworms and aquatic invertebrates [26, 27]. Only three adult midges or two larvae (about 10 mg) were required to quantify carbamazepine (see Section on sample preparation).

Among the stationary phase materials tested for the cleanup of extracts, C18 (non-endcapped) SPE material revealed best results (see Table 1): the signal area was similar for all materials tested and for the raw QuEChERS extract, when spiked before injection. The relative standard deviation in spiked sample extracts was 2.8% (*n* = 5) for signal areas. However, the greatest signal suppression (for calculation, see below) due to interfering matrix components was observed in raw extracts. The highest signal intensity was obtained for the extract cleaned with the combination of both dSPE materials, simultaneously removing polar and non-polar matrix components. The highest signal intensity of 88% and 78% was obtained in the sample cleaned with C18 + PSA or PSA, however, accompanied by the lowest recoveries of 44% and 43% (see Table 1).

**Table 1:** Effects of dSPE materials: comparison of effects of dSPE cleanup with different sorbents on signal intensity and recovery of carbamazepine in adult midge larvae extracts

dSPE step	Signal area in sample + spike [%]^a^	Signal area in extract + spike [%]^b^	Recovery [%]^c^	Signal suppression [%]^d^
PSA	34	78	43	22
C18	50	77	65	23
C18 + PSA	39	88	44	12
Raw extract	46	76	61	24

^a^Signal area in sample + spike is the area detected in samples spiked before the extraction (this includes losses during workup and matrix effects)

^b^Signal area in extracts + spike are extracts spiked before injection (this value only accounts for matrix effects)

^c^Signal suppression is calculated by 100% − extract + spike

^d^Recovery was calculated by signal area in extracts to signal area in extracts spiked after the extraction; details on the calculation are given in the “Data analysis” section

In literature, a comparable signal suppression of approximately 20% was reported for carbamazepine signals in invertebrate extracts cleaned by hexane, but no effect on recovery by C18 + PSA cleanup [26]. As the dSPE step removes matrix components but also some analyte, the lowest limits of detection were obtained when the enhancement of the signal area (due to removing interfering matrix components) was greater than analyte losses during the extraction. The highest overall signal intensities were obtained for carbamazepine after C18 dSPE, followed by the raw extract, although signal suppression was higher than in the other treatments. PSA seemed to remove relatively large amounts of carbamazepine and was thus not considered further. In the final method, the cleanup with C18 was implemented. Filtered medium samples were analyzed directly by HPLC–MS/MS in contrast to literature, where SPE was used [22].

#### Figures of merit

For aqueous samples, the linear range was 0.1–32 μg/L (10 calibration points; *R*^2^ = 0.9996). An LOD of 0.1 μg/L and an LOQ of 0.2 μg/L were calculated using signal to noise ratios of 3 and 10. For biota samples, the linear range covered 1–40 μg/L with a correlation coefficient of 0.9947 for larvae. This range covered typical concentrations in the exposure experiment. Quantification in biota was based on matrix-matched calibration using an isotopically labeled internal standard to correct for losses during sample preparation and instrumental analysis, even if signal suppression occurs [28].

The recovery was acceptable with 95 ± 15% based on the quantification with the internal standard. LOD and LOQ were 1 μg/L (S/N = 3) and 5 μg/L (S/N = 10), comparable to results by Dussault et al. (LOD of 1.1 μg/L; LOQ 2.8 μg/L in biota) [22]. In similar studies, emerging pollutants were extracted from individuals of *Potamopyrgus antipodarum*, *Valvata piscinalis*, and *Gammarus fossarum* and small numbers of *Chironomus riparius* larvae and subsequently analyzed by nano-LC–MS/MS [23, 26, 27]. QuEChERS followed by HPLC–MS/MS was also applied for detection of carbamazepine in earthworms [24] with a sample size of 10 mg.

### Body burden of carbamazepine in larvae and adult midges

**Fig. 1:**
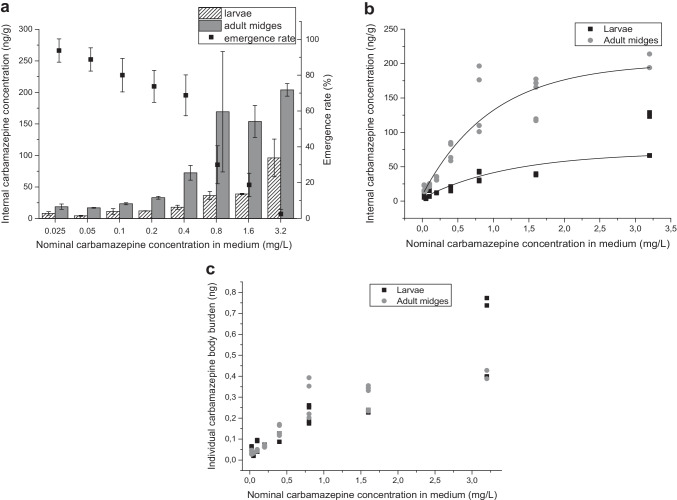
**a** Internal concentrations and emergence rates: emergence rate in % and internal carbamazepine concentration for larvae and adult midges (ng/g wet weight) depending on the nominal concentration of carbamazepine in the exposure medium. Biota samples were extracted with the optimized QuEChERS method and quantified by HPLC–MS/MS in two independent workups (see the “Materials and methods” section). **b**, **c** Comparison of internal concentrations and body burden: **b** Exponential fit of internal concentrations in larvae and adult midge tissue extracts (average values in **a**). The results of the two replicates and two measurements each are given for each concentration level. **c** Body burden of carbamazepine in ng per individual larvae and adult midge, data from **a**, based on the average mass of 2 mg (adult midges) or 6 mg (larvae)

Emergence rates and contaminant burden were clearly concentration-dependent with 82.5 ± 8.0% of adult midges emerging in the control (corresponding to 17.5% larval mortality) but only 2.5 ± 2.9% at a nominal carbamazepine concentration of 3.2 mg/L (97.5% larval mortality) (Fig. 1a). Concentrations ranged from 3 to 129 ng/g (wet weight) in larvae and 14 to 278 ng/g in adult midges. Differences between replicates were low except at 0.8 mg/L nominal exposure concentration for unknown reasons. The adult midges emerging from exposed larvae had a significantly higher internal contaminant concentration in tissue extracts (threefold) at all exposure concentrations. A good correlation between internal concentration and emergence rate is visible from Fig. 1a.

An exponential fit well described the dependence of internal concentrations in tissue extracts on the exposure concentration, see Fig. 1b. The graphs for larvae and midges merged (see Fig. 1c) when calculating the carbamazepine mass per individual (body burden) assuming an average body mass per individual of 2 mg (adult midges) and 6 mg (larvae), as determined in our study.

The bioconcentration factor (BCF) of carbamazepine, the relation of the detected concentration in tissue to the exposure concentration determined experimentally in the middle of the experiment at day 14 of the experiment (63% of the nominal concentration), was 0.09 L/kg for larvae and 0.29 L/kg for adult midges, calculated as a mean value of all data, excluding the lowest concentration being close to the LOD.

### Estimation of the exposure of tree swallows

For the exposure estimate, we used the contaminant concentration in the larvae and adult midges (see Fig. 1b and c), the average weight and energy supply by the adult midges, and the energy consumption and weight of the predator, also considering the digestion efficiency [1]. Miller et al. [15] detected carbamazepine in gammarids at a concentration of up to 6 ng/g dry weight at a surface water concentration of 320–344 ng/L. In our study, an exposure to 25 μg/L water concentration led to a concentration of 8 ng/g wet weight, see Fig. 1b. Carbamazepine was detected in surface waters in the range of 80–800 ng/L [10] and up to 8000 ng/L [11]. For the following discussion, we assumed a water concentration of 1 μg/L. From our results, we estimated a minimal internal concentration at this exposure level of 0.2–0.5 ng/g. Clearly, carbamazepine was not excreted upon pupation. In the adult midges feeding terrestrial predators, a threefold higher concentration was observed (Fig. 1). We thus calculated with 1–100 ng/g (wet weight).

Tree swallows have a very high percentage of insects with an aquatic larval stage in their diet (~ 60%, determined in bolus samples), if their colony is close to a stretch of water with only low seasonal fluctuations [29]. We estimated the uptake of carbamazepine by wild birds using daily energy expenditure [30, 31] for the daily food intake, calculated for wet weight. As an example, Crocker et al. [30, 31] calculated the energy content of arthropods to be 19.6 kJ/g dry weight with a moisture of 77.3%. Therefore, arthropods deliver up to 6.5 kJ/g wet weight. For a chaffinch with a similar weight as a tree swallow of 20.9 g (tree swallows 18–22 g [32]), we can calculate the daily energy required to be 85.2 kJ/day (extrapolation from regression data in [31]). This equals (85.2/6.5) = 13.1 g insects to account for the daily energy demand. When we assume a digestion efficiency (energy recovery) of 75% by the intestinal tract, 17.3 g insects (wet weight) are consumed per day or 10.4 g chironomids (60% chironomids in the diet). Summarizing all data, we calculated the daily uptake of carbamazepine in absolute values and the dose based on body weight (Table 2).

**Table 2:** Exposure estimation: estimated carbamazepine content in chironomid midges (wet weight); daily uptake by tree swallows assuming a daily consumption of 10.4 g chironomids exposed to carbamazepine during their aquatic larval stage. One microgram per liter of water concentration was assumed to lead to 1 ng/g tissue concentration in adult midges

Carbamazepine content in adult midges in ng/g	1	10	25	100
Daily uptake by birds (in ng/day)	10.4	104	260	1040
Daily dose per body weight (in ng/g/day or μg/kg/day)	0.5	5.0	12.4	49.8

## Discussion

### Internal concentrations, bioconcentration factors, and fate upon metamorphosis

Overall, there is only a small body of work considering not only the biological effects but also the uptake of the chemical under investigation in exposure studies. The method presented here is advantageous as it allows quantifying carbamazepine in both larvae and adult midges. Our new analytical method allowed quantifying carbamazepine in about three adult midges or two larvae, so nearly on the level of individuals. This is important to better understand the fate of chemicals during metamorphosis. Only then, bioconcentration factors can be determined with sufficient accuracy.

Emergence rates strongly decreased at higher concentrations, especially at 0.8 mg/L. At low, environmentally relevant concentrations, however, there was no significant effect. This result is in line with the results of Heye et al. [18] reporting EC_10_ and EC_50_ values of 0.317 and 0.677 mg/L. We observed internal concentrations (ng/g) to increase exponentially with water concentration and further by a factor of 3 upon metamorphosis (see Fig. 1a and b). However, considering the parallel reduction of biomass by a factor of 3, the original body burden remained (see Fig. 1c). This finding correlated with the threefold increase in larvae lipid content from 1.5% wet weight in larvae to 3 and 3.5% wet weight for male and female midges [33]. Carbamazepine is of medium hydrophobicity (log *K*_OW_ = 2.25 [34]) and can thus be expected to be stored in fatty tissue. Clearly, for *C. riparius*, carbamazepine is more or less completely transferred during metamorphosis.

The fate of wastewater micropollutants during metamorphosis has not yet been addressed given the rare studies analyzing these compounds in biota. For other classes of compounds, more data are available: PCB concentrations increased between life stages [5, 7, 33, 35] with bioaccumulation factors of 1–5, but 1.8 when referencing to extractable fat [5]. During metamorphosis, 82.6% of the chloropesticide trans-chlordane were retained, while 11.4% were left behind in the shed exuviae [36]. Kraus et al. [7] hypothesized that compounds that are conserved metabolically in insects and thus retained in food webs such as organochlorines would persist during metamorphosis, leading to a higher risk for predators of adult insects. For PAHs with log *K*_OW_ < 5, persistence decreased with hydrophobicity and molecular weight [7]. Carbamazepine with a log *K*_OW_ of 2.25 proved to be persistent.

With a BCF of 0.111 ± 0.06 L/kg for larvae, carbamazepine cannot be considered bioaccumulative (criterion BCF > 100) in *Chironomus riparius* larvae. For comparison, in other aquatic organisms, higher BCF values of 7.1 L/kg in the crustacean *Gammarus pulex* [34] were calculated.

### Aquatic insects as vectors of contamination between ecosystems

Tree swallows are migratory, aerial-feeding insectivorous birds, nesting near rivers and lakes and will become exposed to contaminants in water and sediment via emerging insects. Tree swallows are often used as sentinel species to investigate contaminant exposure across North America [37]; thus, data on their diet are available to estimate their exposure.

Table 2 shows that already at an aqueous carbamazepine concentration of 1 μg/L leading to internal concentrations of only 1 ng/g in adult midges, daily doses of 10.4 ng/day or 0.5 μg/kg/day can be assumed for tree swallows feeding predominantly from insects with aquatic life stages. Over the 6-month summer period, up to 2 μg may be consumed. For elevated water concentrations close to 10 μg/L, even 5 μg/kg/day are calculated. For comparison, the human maximum recommended defined daily dose of carbamazepine is 1000 mg/day (or 14 mg/kg/day assuming a body weight of 70 kg) [1]. Using the same assumptions, citalopram (log *K*_OW_ = 3.74 [38]) with a larval concentration of up to 6 μg/g (dry instead of wet weight used here) in streams [1] would give rise to an exposure estimate as high as 3 mg/kg/day (0.3 mg/kg/day recommended for humans [1]). These data clearly show that aquatic insects are relevant vectors for pharmaceuticals and other wastewater contaminants from the aquatic to the terrestrial ecosystem and have to be considered for risk assessment.

Trophic transfer was studied for different prey-predator scenarios and compounds: e.g., riparian spiders as terrestrial insectivores to assess local aquatic/sediment contamination and fluxing of PCBs and pharmaceuticals between aquatic and riparian food webs [1, 9]. Papp et al. [37] and Echols et al. [12] clearly showed that PCB congener profiles in nestlings of tree swallows were linked to dietary uptake of PCBs (comparing results for nestlings, insects, and food boli). Bioaccumulation was demonstrated comparing PCB concentrations in sediment, emergent aquatic insects, terrestrial insects, and gut content of nestlings of tree swallows [8]. Similarly, the exposure risk for arachnivorous birds was discussed [4].

### Effects of carbamazepine on birds

The effects of carbamazepine on birds have rarely been studied. We presume that this is due to aquatic wastewater micropollutants currently not being considered relevant for the risk assessment. Environmentally relevant concentrations of carbamazepine impaired morphogenesis in a dose- and stage-dependent manner in chicken embryos [39]. Effects on gastrulation, neural tube closure, differentiation, and proliferation were exhibited in early embryonic stages at doses of only 0.2 μg/kg embryonic weight but declined during development. Carbamazepine was suggested for use in pet birds with feather-picking disorder but caused bone marrow suppression and hepatotoxicity [40]. Food competition aggression behavior in pigeons was reduced at oral doses of 20 mg/kg [41].

### Consequences for risk assessment

Contaminant flux may (i) be reduced by loss of prey biomass in case of toxic effects on larvae with lowered emergence rates but (ii) increased in cases of low effects on larval survival and on metamorphosis (Wesner et al. and Walters et al. in [2]). Otterer et al. in [2] would not consider insect-mediated carbamazepine fluxes as an exposure pathway as it proved not to be bioaccumulative, failing in one of four criteria (no/low influence on larvae mortality and emergence rates EC_10_: 317 μg/L [18] (reported maximum concentration in surface waters worldwide: 8 μg/L [11]); contaminant retentions through emergence).

However, so far, all advices on risk assessment are based on data for metals, PAHs and PCBs, and chlorinated pesticides ([42] and Otterer in [2]) but not for pharmaceuticals which are not present in the terrestrial habitat. Accordingly, very high bioaccumulation factors would be calculated for terrestrial predators, which could not develop adaptation strategies. In addition, being designed as active substances, severe effects on higher trophic levels are more likely.

## Conclusion

Our results clearly show that the insect-mediated contaminant exposure can be significant and may even increase during drought periods and upon climate change where contaminant concentrations in rivers increase despite constant wastewater influx. This becomes critical, when temporal linkages with emergence and nesting occur. Our results indicate that terrestrial predators may become chronically exposed to many different wastewater micropollutants, which are kept in the insect upon metamorphosis as observed for carbamazepine. The results for carbamazepine show that already with a relatively low bioconcentration factor, significant doses may be reached. However, the development of analytical methods for biota analysis has to be intensified to aid risk assessment. In addition, the investigation of sediments for micropollutants is needed given that many aquatic insects have benthic life stages.

## Data Availability

All data are included in the manuscript.
